# Address to the Graduates of the Medical Department of the University of Buffalo

**Published:** 1854-04

**Authors:** Thomas F. Rochester

**Affiliations:** Professor of the Principles and Practice of Medicine; Buffalo


### BUFFALO MEDICAL JOURNAL

AND

### MONTHLY REVIEW.

**VOL. 9.** APRIL, 1854. **NO. 11.**

### ORIGINAL COMMUNICATIONS.

ART. I.—*Address to the Graduates of the Medical Department of the University of Buffalo.* By THOMAS F. ROCHESTER, M. D., Professor of the Principles and Practice of Medicine. Feb. 22d, 1854.

Those who have devoted themselves to the beautiful study of the Natural History of Insects, tell us that the Mother Bee, the Queen, whose subjects are, *in truth,* her children, deposits, with unerring precision, in their respective cells, the ova which produce the three orders in which her community is divided — that these ova undergo several metamorphoses, the first change being into that of larvæ, the next into that of pupæ or chrysales, and the last into that of the perfect insect. They tell us, moreover, that these *changes* are the result of what may be termed the instinctive volition of the insects who have a temporary charge of those who are to constitute the next generation — that according to the FOOD that is given to the larva, it will, or will not, eventually be a worker.

What the insect does for the embryo, the instructor does for the pupil. He selects and prepares the aliment that is to form, strengthen and guide the mind — how weighty, how responsible, and yet how grateful is his office. But while he would instruct his pupil, he must, also, school himself. He is to teach fact, not fancy — sound reason, not sophistical speculation — truth, not error.

How vast, how wondrous, how great is *science!* It is not measurable by space, for it is infinite, limitless. For what is science? It is "knowledge" —"certainty based upon demonstration"—"art built upon principles." Alas! that man, science' sole votary, should be so weak, so finite and so brief. Man physical, *is* weak; he appears for a moment, distinct, embodied, among other atoms, and then is swept away forever, by one of the great billows that Time is forever pressing on in surges. But this is not true of man's Mind—this, the exclusive and distinctive gift of the All-Powerful to him whom he created "after his own image," bears the impress of Divinity in its extent, its power, and its achievements. Mind has ever grown, and will grow, while mankind exists—it is transmitted from generation to generation, not individually alone, not from parent to offspring solely, but through the succeeding generations of the whole vast human family. Now, science is the mind's great pabulum—its food; and of this food every liberal profession furnishes its quota—and should not ours be large? We take no stinted handful from its stores—should we not return measures heaped up and overflowing?

This day, gentlemen, you are received into the ranks and fellowship of the Medical Profession. I congratulate you upon your new and honorable position. It has been the earnest, and, I trust, the evident and successful care of my colleagues and myself, to place before you that proper and sufficient intellectual aliment that guides and stimulates the recipient to correct and constant mental culture. We cannot make you *surely* always workers, but we hope we have established in you a wish and a will to be such. In this day and generation, work you must, or you will make no mark—you will be counted with those who have lived for naught, or rather counted not at all. In ceasing to be pupils, you must not cease to be students—your degree is a staff to guide your advancing steps, not a support to sustain and bear your *weight.* Now indeed is the time for you to commence your lifelong task, for a medical man must be a student alway. By his profession he is the declared authority upon all matters of public and private hygiene. He is not only to relieve and restore those who are ill, but he is to point out to those who are well, how they may secure and preserve that priceless jewel—Health. It is unnecessary for me to dwell upon the amount and variety of knowledge that such a position involves, or how constantly it demands additional and more varied stores. Happy are you, gentlemen, in the epoch, and in the field in which you are about to begin your investigations. "Young Physic" and "Young America" should go hand in hand—they will journey well together.

Young Physic! Young America! these are glorious titles, and pregnant with a mighty future. And yet as terms, as mere names, they are not without a stigma; but that is because they are confounded with the mass of vapid, empty, clamorous cheats, that hang about, and strive to attach themselves to the rising giants. As the strong tower ascends, rubbish and refuse accumulate about its base; but the proud structure completed, these are all swept away, and it stands erect, alone in its majesty and beauty.

Young Physic, in name, is a new creation—in reality it is the modern medical mind, the legitimate offspring of a lawful parent, but less trammeled than its predecessors by the rules and laws of a code somewhat too rigid. Born in an eventful and stirring age, an age of unequaled progress and discovery, it could not but catch the spirit of the times; and stimulated by that, it rushes on with the impetuous current, and being no laggard but a brave bantling, what wonder that it should now be found among those of a kindred spirit who keep upon the crest of the topmost wave.

It denies not the parental authority. It is not ignorant of the ancestral code. It acknowledges and respects them both, but it does not feel bound to endorse all their dogmas—it claims the right of election. It has a mind of its own, and chooses to submit everything to its scrutiny. It requires, as far as may be, demonstrative evidence; for by the many new lights of science, much is now demonstrable as false or true, that before, of necessity, rested mainly upon theory.

Young Physic is not, therefore, unmindful of the past, but it does not *cling* to it—it looks rather to the present and to the future, and bold is its glance and far its ken. Of authorities, it constitutes itself the judge, esteeming none as such upon mere repute; it brings all to the test, and by the same trial does it receive, or reject them.

Such is Young Physic—the true and not the counterfeit. He is now beginning to display his power, and already are trembling beneath his frown, the motley and multiform crew—the successive progeny of Charlatanism and quackery—that under various names, and with the cant use of the terms "reason," "philosophy" and "liberty," (the gabble of all imposters) have endeavored to palm themselves off as his associates—nay, even as his veritable self.

Young America! We take it in its geographical and in its political sense.

In the former we see a vast country extending from the clime of the long winter-bound north, to that of an ever-summer south, and from east to west holding apart the two great oceans by a barrier more than 2000 miles in breadth. And can we say that the limits are yet fixed? Are they not rather to be bounded by the same lines as the whole western continent? By Young America *political,* I mean not that class of modern fillibustieros and demagogues, who are justly defined by one now adorning our legislative councils, as those "who hold nothing sacred in the past, who oppose on principle or passion all conservatism, and run rampant over all institutions which interpose barriers to the attainment of their ends." No. I mean a young, a healthy, a national, self-relying, and self-defending spirit—a spirit of independence, of progress and reform.

How often is it said by foreigners of Americans—they are a people, but not a nation—they have a government, but they have no distinct nationalism—they are essentially composite; they spring from European ancestry, and though the blood-stream is much mingled and confused, still, the rejected parent stock controls taste, fashion, and education. In short, they assert that as a people, the Americans have peculiarities, but not characteristic national traits. The time for, and the aptness of this assertion are passing away. We are establishing, nay, we have established, a nationality. We are creating customs, and habits, and modes of our own. We have partly ceased to borrow and imitate from the old world, and those who come to us from it, fall into our practices and uses. Young America is rising and rejoicing in her strength. An Americanism is no longer of necessity a provincialism, and a true-hearted American will not hesitate to maintain an act, or an expression, *because* it is American. It has been said that as a people we are over-sensitive, and that our susceptibility to ridicule on minor matters, exposes a weak point that will ever be taken advantage of. The remark is just—and it has been further, and no less truly said, that this susceptibility is the result of slight and imperfect education. Now this latter is a notable and grievous fault, and we, as members of a liberal profession, should be among the first to see, to acknowledge and to remedy it. We boast, and justly, of our common schools, and say with pride that none need be totally ignorant; but we should also admit that there are but few, very few, scholars among us.

I have spoken of "Young Physic" and "Young America," and I have told you what I understand them to be; and I have brought them forward together, because I wish to speak of the natural advantages that America affords to the student, and of the field that lies before him, if he will use and improve those advantages. And by student, I of course here allude to one who devotes himself to medical science, although similar advantages are equally patent to the inquirer in any department.

And firstly, let us consider, the extent of our country. Its broad geographical limits afford ample opportunity for investigating the influences upon man, of climate, of temperature, of mountain, midland, and of lowland, of city and of country, of seaboard and of inland residence, with all their variety of soil, vegetation, and other local peculiarities: and the investigations may be more accurate, and the deductions more correct, than those instituted elsewhere over a corresponding extent of clime and surface, because they will apply more closely to one and the same people, leading in the main the same mode of life; for as yet, the great majority is of the Anglo-Saxon race. Nor should our observations be limited as to the influence of locality upon the production of endemic disorders. The course and character of various epidemics is unquestionably modified by peculiar conditions of soil, of climate and of atmosphere. Nature opposes to certain scourges insurmountable barriers, which, to the advance of others, present no obstacle. Some pests she seems to invite, and to encourage them to linger in devoted spots. The epidemic cholera has not *confined* its march to miasmatic districts, but it has made long halts among them. How has it tarried about our western waters, and how loath is it ever to leave those rich bottom lands, that, lovely and inviting in appearance, and possessed of a garden-like fertility, are yet deeply tainted with the paludal poison. But if in this vast extent of varied territory there is a corresponding diversity of diseases, or rather, perhaps, of phases of disease, there are likewise products of nature with which man may combat the agents of the destroyer, and with most of these we are, as yet, unacquainted. Of how many of the countless plants indigenous to our soil, do we know the medicinal properties? Of very few. For the great bulk of our materia medica—our armamentum medicum—we are dependent upon the old world. This should not be so. We have, doubtless, more potent weapons within our reach, and we should strive to learn their uses. The American Medical Botany has yet to be written. No enthusiast can complain of the narrowness of the field.

Our mountains and our valleys abound in chalybeate and other mineral springs, and although their waters have generally been analyzed by competent persons, yet much remains to be done in the way of exact and proper examination as to their administration and application in disease.

The thoroughly educated physician should properly be no unskilled naturalist, and our streams and forests have many inhabitants, thus far undescribed and unclassified. The science of Comparative Anatomy is yet to receive many contributions from them: and I trust ere long, that savans from abroad, while they instruct and delight us, will not make us feel how sadly ignorant we are upon this subject.

These are a few, and but a few, of many matters requiring more complete investigation than has yet been devoted to them; and I revert again to the unusual inducements for observation, taking into account the extent of territory, that our country affords. These are briefly:

1st. Greater correctness of deductions, the observations being made upon one people.

2d. Ease of acquiring information from unity of language.

3d. Small amount of necessary pecuniary expenditure.

4th. Facilities of travel, both by land and water, afforded by what, without a suspicion of national conceit, I may justly and exultingly speak of as the great American agent of locomotion—steam.

Lastly, for I think it may be spoken of in this connection, the establishment, as a permanent institution, of the American Medical Association, which gives every opportunity for the elucidation of that which may be obscure, for the correction of that which may be erroneous, and for the chronicling and diffusion of that which is found worthy and original: for it is the sole object and aim of this body (whose members are from every section of the Union) to promote and sustain among medical men, a proper and professional spirit.

The inquiry now arises: are our republican institutions favorable to the pursuit of medical science? In their general effect they are, beyond a question, although it is a grievous truth that neither our federal or state governments give that encouragement to general or special science that a liberal and enlarged policy dictates. In this respect they are far behind all other enlightened authorities. But the advantages to be derived from such encouragement, are more than counterbalanced by the fact that, if there is no direct support, there is no indirect impediment. Here, and here alone, man may make his way from the lowest to the highest position—he is secure in the enjoyment of his civil and religious rights—no surveillance puts a curb upon his tongue, a clamp upon his movements; he is free to express his honest convictions, or to go where his inclinations or his pursuits may lead him. It is this that makes the land of his adoption so dear to the philosopher of Neuf-chatel. It is this that is giving to us as brothers, no longer only the poor peasants, but the educated men of Europe.

It is from no boastful spirit that I have thus laid before you the attractions and the opportunities for scientific investigation that in my view are specially afforded by the Great Republic of the western world. I do not wish in a false or presumptuous manner, or with exaggerated expressions, to expose myself to the charge of hyperbole, by unduly magnifying the favored land in which, thank God, it was my good fortune to be born; but I *do* wish its merits to be understood and appreciated, and its reputation to be established and maintained; nay, more than that, to be constantly exalted. In the scale of nations, in power, in wealth, and in successful enterprise, we acknowledge no superior; and I trust that ere long, with equal right and truthfulness, we may take as proud a stand, and support as just a claim for our professional and scientific men, for our institutions of learning and art. And I wish to urge you, gentlemen, with the most earnest entreaty, to do your part toward effecting so desirable a consummation; indeed, it is an imperative duty, a natural obligation that you, and I, and all of us—that every one who claims to be a good citizen—owes to the soil of his birth or of his adoption. Let this be the field in which to display your patriotism; opportunities for the manifestation of the most dauntless valor will not be wanting, (and this is especially true in the profession of our choice;) and if you are ambitious of present or of posthumous fame, surely more glorious is the reputation of the soldier who most successfully defends his fellows against disease and death, than the renown of the warrior who shall have sent the most of his kind into the premature power of the great destroyer. But it is not for the establishment of your individual fame, for mere selfish ends, that I wish you to work—aye, that is the word, *work*—but for the renown of your country, and not less than that for the true amelioration and protection, not of your fellows only, but of all mankind. And as the first and most important step in this great and good cause, strive to acquire—and let your efforts cease with life only—a thorough and complete acquaintance with those subjects that pertain to your profession; and, as far as it relates to medical men at least, let it no longer with truth be reproachfully said—that the Americans are imperfectly educated.

I have told you that when you receive your degree, that although that terminates your pupilage, you must not cease to be students—the man who counts upon flinging aside his books on the reception of his diploma, is unworthy of the fraternity in which he is enrolled. If he thinks that in three short years he has acquired a life-stock of all necessary information, he admits that his brain is of most limited capacity. If he is aware of how much he has to learn, and does not strive to obtain further knowledge, but is content to lead a life of mental sloth, and to pursue his practice under the authority and protection of the lettered parchment that certifies that he has satisfactorily undergone an examination in the rudiments of his profession, he is a willful and conscience-void impostor, who prostitutes a noble and sacred calling, filling his pockets and maintaining his own worthless life, at the expense of suffering humanity, receiving as a compensation, fees, false dues for which no equivalent has been rendered. And yet, how often are men heard unblushingly, nay boastfully, declaring that they read no books, save the book of nature, and that they owe their knowledge of disease and remedies to observation,—covering their own ignorance and effrontery by pandering to the popular notion, that the diligent student and scientific physician, cannot be a judicious and successful practitioner. Out upon such fellows! their "observations," their "knowledge" and "practical good sense." Seeing is not observing—their "knowledge" is conceit—and their "good sense" impudence or stupidity. It is of such stuff and fustian that quacks, in and out of the profession, are made. Can there be greater presumption than that one man should set up his "experience" against the combined judgment and real observation of earnest and studious men, whose opinions are formed upon principle and whose investigations are conducted with care and precision, and upon a scientific basis? who meet to declare and to discuss their several views, and who publish their ideas and discoveries without reserve, and not for the purpose of notoriety and self-glorification, but for the benefit of their professional brethren, and through them for the benefit of all mankind? I have not thus spoken, gentlemen, simply to vindicate the scientific and studious physician, and to protest with all my energy against the false, outrageous and wicked assertion that is so often made by pseudo-physicians, and by others that are uninterested, and who, for their own welfare, should be disabused of this pernicious and prevalent notion. I have not, I repeat, spoken for this object alone, but I also wish to persuade you who are just entering upon the course, to take your first steps aright—to pursue your profession as a science and an art, and not as a trade—for as a mere mechanical vocation, it can have no rank, and should be classed below the humblest manual calling whose followers may be skillful and finished workmen, honest men and good citizens.

And now, gentlemen, that I have told you what is wished and hoped for from you, it will, perhaps, be expected that I should map out certain lines, or traces, by whose assistance, or guidance, you may arrive at the desired position; but this I cannot undertake; in one whose years in the profession are so few as mine, the act would be presumptuous. But I may refer you both for precept and example, to those gentlemen with whom it is now my pride and pleasure to be associated as a collaborator in the institution in which you have been pupils, and from the lips of no less than four of whom, I am glad to have and to embrace this opportunity to state, it was my good fortune to receive my first instructions in medicine. Where, I may proudly ask, can you see more strict regard to professional etiquette, associated with firm and uncompromising opposition to every form of extra or intra-professional delusion or empiricism? Where more happily illustrated when combined with the highest professional skill, the success of perseverance, energy and determination, with unshaken firmness in maintaining the right and opposing the wrong? Where more honesty and candor, with intrinsic evidence of superiority, than that which is manifested by the publication of tables and statistics, that have already shielded more than one unfortunate man whose skill and attention have been repaid by malice and ingratitude, because a fractured limb, which, but for him, perhaps, would have been lost altogether, has been restored to use, but in symmetry not quite the fellow of that which had not been injured; a result that these tables demonstrate to be in every such instance almost inevitable.

Where will you find the wonders of the laboratory more successfully displayed, than by one whose thorough and varied knowledge comes forth the more brightly and distinctly, because relieved by the shade of a quiet and retiring deportment? Where *elsewhere* in our whole land will you find the mysteries of animal life and action elucidated, explained and presented, in situ, in movement and in function, while no pang of anguish afflicts the animal reposing in a happy and complete unconsciousness induced by an agent whose anæsthetic properties were discovered and employed first in America.

Where may you look for more complete anatomical knowledge and surgical dexterity, than that which calls from a large practice its possessor to give instruction in each department in two institutions, and whose services a third sought in vain to retain?

Are not these examples to encourage and guides to follow? and is it not right that I should here, and on this occasion, speak proudly of them; and when I seek for exemplars to hold forth to you, because I find them in my colleagues, should I keep silence? nay, should I not rather express the great satisfaction that it affords me to be able, in all honesty and sincerity, to make the selection where my preferences would naturally incline?

I have disclaimed the attempt, gentlemen, to lay down any certain or fixed rules for your guidance and direction in the path you are about to follow; but perhaps a few hints and suggestions as to what, in my opinion, is the proper course, may not be unacceptable. Many of you will enter at once upon the active duties of your profession; some in the open country, some in the flourishing village, some in the crowded city, some, perhaps, in hospitals or other public charities, and some of you, as I am informed, will visit those old and extensive institutions in Europe, that justly attract to them inquirers, students, from all portions of the enlightened world. Many of you, for a few years at least, will have abundant leisure, and none will be so exclusively occupied that they will not have more or less time at their command; and this time it is your duty, and will prove your interest, to spend in study, thought, and scientific investigation. As a primary and essential feature, establish a system of order in your pursuits. Without neglecting that general reading that the various diseases of your patients may render obligatory, take up, *seriatim*, those subjects that are connected with important specialities, and render yourselves as far as possible conversant with their principles and details. It may be that you will detect and point out errors, or that you may be able to add useful information to the knowledge already possessed, and thus from simple students, your investigations may enable you to become discoverers and instructors. But let me impress upon you the necessity of thoroughly studying one subject before you take up another or others. Many a really fine intellect has been wasted by the indolence and fickleness of its possessor, who, in attempting to deceive others and perchance himself, by a show of extended and diversified acquirements, has subjected himself to exposure and to merited mortification and chagrin. I would advise you by all means to keep a careful record of your practice. The labor in prospective may seem great, and in many instances useless, but it will not prove so: observations at one time apparently trivial, may ultimately prove important. In this exercise facility will be soon acquired, and it will contribute greatly to give system and point to your inquiries. Your aim should always be, to combine perspicacity with conciseness, omitting nothing that is useful, but avoiding needless detail. From this record you will do well to select cases for publication, if you judge, upon examination and reflection, that they will convey useful or novel intelligence; and let the observations with which you may see fit to accompany them, be pertinent to your subject. In your language, eschew alike pedantic technicalities and every approach to slang and vulgarism. And lastly, be careful in the selection of the Journal to which you entrust your communications—choose one that is conducted with ability, and that maintains a high and honorable position.

A knowledge of medical microscopy, although not absolutely essential to a respectable professional standing, is an acquirement without which at this day, one can scarcely lay claim to a finished education. The expense of a proper instrument will prevent many of you from procuring one immediately, but the outlay that is absolutely necessary, is not in reality very great, and a little economy will soon afford the requisite sum. To those of you who are able to do so, I would say, purchase, and begin to use this instrument at once. It is true that its revelations are distrusted and derided by some, but they are of the same class of contracted bigots that held fallacious or made sport of the immortal discovery of Laennec. The aid afforded by physical exploration in determining the condition of certain structures, is no greater, nay, perhaps it is less reliable, than the positive information presented by the microscope. The latter enables the surgeon to distinguish readily between disease that is (technically speaking) malignant or benign. It tells the physician the changes the various excretions and secretions have undergone. It exposes to the pathologist the most minute structural lesions; and it confirms to the physiologist, the assertion of the sacred volume, that man is "fearfully and wonderfully made." Can we then regard as a mere accomplishment, the information to be acquired by this method of research?

But, gentlemen, I must not longer occupy your time and that of the friends who have assembled to do honor to this occasion. I have attempted to urge you to that course of conduct that in my humble judgment will do credit to your country and yourselves. Those of you who may go abroad will see that each great country has institutions that are peculiarly national. There are French, English, and German schools of medicine. Let us do our part in forming what shall be emphatically and distinctively the American school. If we receive instruction from others, let us make it worth their while to seek it also from us.

I have said nothing of the social and moral relations that your profession entails upon you—*that* would surely be needless after the able and eloquent discourse that you have so recently heard from the learned divine, who has this evening implored for you and for all of us, the blessing of Him who is all-powerful to save or to destroy, and who, as becomes us with grateful hearts to remember, has not permitted death to take one from our number.

With regard to your intercourse with your professional brethren and with your patients, I have only to add—be honest and true. Maintain your own self-respect, and you will secure the regard and confidence of your fellows.

Gentlemen, may your future be bright and honorable. In behalf of the Faculty of the Medical Department of the University of Buffalo, and with their earnest wishes for your success and happiness, I bid you farewell.

BUFFALO, Feb. 22d, 1854.

